# Lamb Wave-Based Damage Fusion Detection of Composite Laminate Panels Using Distance Analysis and Evidence Theory

**DOI:** 10.3390/s25185930

**Published:** 2025-09-22

**Authors:** Li Wang, Guoqiang Liu, Xiaguang Wang, Yu Yang

**Affiliations:** 1 State Key Laboratory of Mechanics and Control of Aerospace Structures, Nanjing University of Aeronautics and Astronautics, Nanjing 210016, China; 2 National Laboratory of Strength and Structural Integrity, Aircraft Strength Research Institute of China, Xi’an 710065, China

**Keywords:** Lamb wave, signal features, distance analysis, evidence theory, composite laminate panel, probability of detection, false alarm rate

## Abstract

The Lamb wave-based damage detection method shows great potential for composite impact failure assessments. However, the traditional single signal feature-based methods only depend on partial structural state monitoring information, without considering the inconsistency of damage sensitivity and detection capability for different signal features. Therefore, this paper proposes a damage fusion detection method based on distance analysis and evidence theory for composite laminate panels. Firstly, the signal features of different dimensions are extracted from time–frequency domain perspectives. Correlational analysis and cluster analysis are applied to achieve feature reduction and retain highly sensitive signal features. Secondly, the damage detection results of highly sensitive features and the corresponding basic probability assignments (BPAs) are acquired using distance analysis. Finally, the consistent damage detection result can be acquired by applying evidence theory to the decision level to fuse detection results for highly sensitive signal features. Impact tests on ten composite laminate panels are implemented to validate the proposed fusion detection method. The results show that the proposed method can accurately identify the delamination damage with different locations and different areas. In addition, the classification accuracy is above 85%, the false alarm rate is below 25% and the missing alarm rate is below 15%.

## 1. Introduction

Based on advanced sensing networks embedded on the structure surface, aircraft structural health monitoring technology can first acquire the monitoring signal regarding the structural healthy state. Then, the signal features representing the structural healthy state are extracted with load analysis methods and signal processing methods. This technology shows enormous application potential in improving structural safety, reducing maintenance costs, performing the predictive maintenance strategy and prolonging the service time [1,2]. According to the diversity of sensing networks, the structural health monitoring (SHM) technology can be divided into piezoelectricity transducer (PZT) [3,4], fiber Bragg gating (FBG) [5], acoustic emission transducer (AE) [6] and comparative vacuum monitoring (CVM) [7], et al. [8,9]. With the merits of long propagation distance, low signal attenuation and high sensitivity to the small damage of Lamb waves (LWs) actuated by PZT, the damage monitoring technology using LWs has been regarded as an effective and appealing damage monitoring technology.

According to the structural damage monitoring capability using LWs, the damage monitoring technology can be classified as damage identification, damage location [10] and damage quantitative [11], among which damage identification is the most basic and essential research domain. Many damage identification methods have been developed. Liu et al. [12] proposed a damage identification method based on the energy ratio damage index (*EDI*) using LWs and Hilbert transform and validated on the damage evolution experiment of composite lap joint specimens. The results show that the threshold of *EDI* is capable of identifying the disbonding damages of composite lap joint specimens. Shahab et al. [13] extracted twelve signal features from time and frequency domains perspectives and compared the debonding damage detection capacity of different features. Wu et al. [14] extracted the energy ratio features of time, frequency and time–frequency domains and compared the damage detection capacities using damage imaging methods. Su et al. [15] developed a Lamb wave-based quantitative identification method of delamination damage using an artificial neural network (ANN), in which Digital Damage Fingerprints (*DDFs*) extracted from the LWs in the time–frequency domain were used as the input for a multi-layer feed-forward ANN under the supervised training of an error back-propagation (BP) algorithm. Torkamani et al. [13] introduced an innovative time-domain damage index called the normalized correlation moment (*NCM*) based on local statistical features of the wave form, which shows a superior capacity on the delamination damage detection and damage assessment compared with *SDCC*. Yan et al. [16] extracted the local time–energy density feature with the Gabor wavelet basic function and took the difference coefficient between the features as the damage index, which detected the simulated damage of composite stiffened panels. Loendersloot et al. [17] introduced fifteen signal features and developed a graphical user interface to visually assess the damage detection performance of different signal features, considering the different damage identification capacities for different signal features. Damage identification using LWs is generally realized by establishing the mapping relationship between the single signal feature and the structural healthy state based on the signal feature threshold or the state equation. However, different signal features present different sensitivity to the damage, unequal damage identification capability and inconsistent damage identification accuracy.

Recently, with the rapid development of multi-source information fusion (MSIF) [18], it has been widely used in the structural damage monitoring field. Christoph et al. [18] reviewed SHM methods based on multi-sensor data fusion for the damage assessment of metal and composite structures and discussed data-level fusion methods, feature-level fusion methods and decision-level fusion methods. He et al. [19] developed a damage identification method for the unmanned aerial vehicle structure, by fusing the strain data, the acceleration data and the modal frequency data with data-level fusion, feature-level fusion and decision-level fusion. Qiu et al. [20] proposed a crack propagation monitoring method based on a guided wave–Gaussian mixture model (GW-GMM) by using LW-based feature extraction to obtain multi-dimensional damage indexes in time and frequency domains and adopted principal component analysis (PCA) to reduce the dimensions and extract the prominent signal features, in which PCA is a feature-level fusion method. Ziemowit et al. [21] developed a damage detection method with some damage indexes as inputs of ANN, which works as a feature-level fusion method. Jiang et al. [22] proposed a multi-sensor data fusion fault diagnosis method based on support vector machine (SVM) and evidence theory, in which one-versus-one multi-class SVM is used to obtain the basic probability assignment (BPA), and the matrix analysis is presented to solve the calculation bottle-neck problem of evidence theory in decision-level fusion. Liewellyn et al. [23] proposed a reliable impact detection strategy for composite structures. In this method, ANN is firstly used as a pattern recognition and classification method with the input of a combination of instantaneous frequencies, continuous wavelet transform (CWT) coefficient integrals, power spectral density (PSD) integrals and Bayesian updating (BU). Then, the Kalman filter (KF) is adopted as a decision-level fusion method to fuse these damage detection results on sub-networks considering the fault sensors network. Yang et al. [24] developed an integrated damage identification method based on the least margin for composite structures. In this method, the identification results of some machine learning models are integrated with the most confidence, which is a decision-level fusion method. However, the research on an LW-based damage identification method with MSIF has not yet been widely studied. And the current damage fusion identification methods based on LW have not considered the strong correlation between the multi-dimensional signal features that have been input, and information redundancy may exist.

Considering the aforementioned challenges existing in both the conventional damage detection methods based on signal features and the current damage fusion detection methods based on multi-level fusion strategy, a damage fusion identification method based on distance analysis and evidence theory is developed in this paper, to obtain the consistent damage identification result for the delamination damage. Firstly, the common 15-dimensional signal features of LWs are extracted from the time, frequency and time–frequency domains. Secondly, the four orthogonal and highly sensitive signal features are retained based on Pearson correlation coefficient and cluster analysis. Thirdly, the data are divided into the training dataset and the testing dataset, whose labels are determined according to the delamination area. Fourthly, the damage identification results and the corresponding basic probability assignments (BPAs) of each highly sensitive signal feature for each testing sample are obtained based on distance analysis. Lastly, the consistent damage identification result is acquired by fusing the BPAs of four sensitive signal features based on Dempster fusion criterion of evidence theory. The accuracy and reliability of the proposed method are validated on damage monitoring experiments of ten composite laminate panels.

The structure of this paper is organized as follows: Section 2 introduces the proposed damage identification method based on distance analysis in detail, including Lamb wave-based multi-dimensional signal features extraction; feature dimension reduction based on Pearson correlation coefficient and cluster analysis; a damage identification-based distance analysis algorithm; a damage identification process based on highly sensitive features and a distance analysis algorithm. Section 3 presents the proposed damage fusion identification method based on distance analysis and evidence theory in detail, including a brief review of evidence theory, BPAs based on Euclidean distance and the whole fused damage identification process. In Section 4, validation experiments on ten composite laminate panels are performed to evaluate the damage identification accuracy and reliability of the proposed method. Conclusions are given in Section 5.

## 2. Distance Analysis

### 2.1. Lamb Wave-Based Signal Features Extraction

In order to directly express the effect of structural damage on LW signals, many typical signal features based on LWs are extracted. Multi-dimensional signal features can be extracted from LWs in the time domain, the frequency domain and the time–frequency domain [17]. In this paper, the common 15- dimensional signal features are extracted, as shown in Table 1.

### 2.2. Feature Dimensions Reduction Based on Pearson Correlation Coefficient and Cluster Analysis

Considering the extremely strong correlation between several features, it is crucial to analyze the correlation of 15-dimensional signal features. Many correlation analysis methods are applied based on the Pearson correlation matrix, the covariance matrix and the multivariate regression model, etc., [25,26,27]. The Pearson correlation matrix is used to reduce the dimensions of fifteen signal features to retain the orthogonal and highly sensitive signal features in this paper.

Assuming that the **X** is multi-dimensional signal features vector shown in Equation (1), in which *κ* is the number of experimental situations, and *a* is the number of feature dimensions, setting to *a* = 15 in this paper.

(1) $$X=\left[\begin{array}{c} X_{1}\\ X_{2}\\ \ldots \\ X_{r}\\ \ldots \\ X_{a} \end{array}\right]=\left[\begin{array}{cccccc} X_{11} & X_{12} & \ldots & X_{1o} & \ldots & X_{1\kappa }\\ X_{21} & X_{22} & \ldots & X_{2o} & \ldots & X_{2\kappa }\\ \ldots & \ldots & \ldots & \ldots & \ldots & \ldots \\ X_{r1} & X_{r2} & \ldots & X_{ro} & \ldots & X_{r\kappa }\\ \ldots & \ldots & \ldots & \ldots & \ldots & \ldots \\ X_{a1} & X_{a2} & \ldots & X_{ao} & \ldots & X_{a\kappa } \end{array}\right]$$

The Pearson correlation matrix **P** is defined as follows:

(2) $$P=\left[\begin{array}{cccccc} \rho_{11} & \rho_{12} & \ldots & \rho_{1r} & \ldots & \rho_{1a}\\ \rho_{21} & \rho_{22} & \ldots & \rho_{2r} & \ldots & \rho_{2a}\\ \ldots & \ldots & \ldots & \ldots & \ldots & \ldots \\ \rho_{r1} & \rho_{r2} & \ldots & \rho_{rr} & \ldots & \rho_{ra}\\ \ldots & \ldots & \ldots & \ldots & \ldots & \ldots \\ \rho_{a1} & \rho_{a2} & \ldots & \rho_{ar} & \ldots & \rho_{aa} \end{array}\right]$$

where *ρ_ra_* is the Pearson correlation coefficient between the signal features vector **X***_r_* and the signal features vector **X***_a_*, and is expressed as follows:

(3) $$\rho_{ra}=\frac{\sum_{o=1}^{\kappa }\left(X_{ro}-\bar{X_{r}}\right)\left(X_{ao}-\bar{X_{a}}\right)}{\sqrt{\sum_{o=1}^{\kappa }{\left(X_{ro}-\bar{X_{r}}\right)}^{2}\sum_{o=1}^{\kappa }{\left(X_{ao}-\bar{X_{a}}\right)}^{2}}}$$

where $\bar{X_{r}}$ is the average of the signal features vector **X***_r_*, and X*_r__o_* is the signal feature under different experimental situations. $\bar{X_{a}}$ is the average of signal features vector **X***_a_*, and X*_a__o_* is the signal feature under different experimental situations.

The sensitivity **Z** is adopted to evaluate the sensitivity of different signal features with the change in structural health states [28]. The sensitivity of the signal features vector **X***_r_* can be expressed as Z*_r_* and can be written as follows:

(4) $$Z_{r}=\frac{\left|\bar{X_{r1}}-\bar{X_{r2}}\right|}{\sqrt{\frac{S_{X_{r1}}^{2}}{p_{1}}+\frac{S_{X_{r2}}^{2}}{p_{2}}}}$$

where $X_{r1}$ and $X_{r2}$ are the signal features vector under the undamaged and damaged states. $S_{X_{r1}}$ and $S_{X_{r2}}$ are the standard deviation of the signal features vector under the undamaged and damaged states. *p*_1_ and *p_2_* are the corresponding number of undamaged and damaged samples.

A cluster analysis algorithm is used to further subtract the feature dimensions. The procedure of the cluster analysis algorithm is as below.

(1) Set the first signal features vector **X**_1_ as the initial cluster set *C_r_* (*r* = 1);

(2) Calculate the Pearson correlation coefficient *ρ_mn_* between each signal feature X*_m_* in the initial signal feature vector set C*_r_* (X*_m_*∈C*_r_*) and the other signal features vector **X***_n_* (*n* = 1, 2, 3…15 and *n* ≠ *m*);

(3) Compare the Pearson correlation coefficient *ρ_mn_* with the threshold *δ*. If the Pearson correlation coefficient *ρ_mn_* exceeds *δ*, then add the signal features vector **X***_n_* into the cluster C*_r_* (C*_r_* = C*_r_∪***X***_n_*). Otherwise, choose the random signal features vector which is not in the cluster C*_r_* as the next new initial cluster *C_r_*(*r*←*r* + 1), and then skip to step (2);

(4) After the 15-dimensional signal features are all added into clusters, count Z of every signal feature in each ultimate cluster under different structural health states;

(5) Select the signal feature with the maximum Z in each cluster and then obtain the highly sensitive signal features set.

### 2.3. Damage Identification Based Distance Analysis

Distance analysis is a lazy supervised learning algorithm, which only saves the samples during the training process without training. After receiving the testing samples, the main training process is as below.

(1) For baseline signals and current signals under different structural health states, the 15-dimensional signal features are extracted and then the highly sensitive signal features are reduced to form the training dataset.

(2) According to the sample labels for each highly sensitive signal feature, the training dataset is allocated into diverse clusters, whose respective label is consistent with the sample labels. In this paper, the training sample label is either undamaged or damaged. Thus, the training dataset of every highly sensitive signal feature is divided into two clusters, one tagged with the undamaged label and the other tagged with the damaged label, defined, respectively, as the undamaged cluster and the damaged cluster.

(3) When the new monitoring signal is acquired, the highly sensitive signal features can be extracted and regarded as a testing sample. For each testing sample, its state label’s predicted result depends on the cluster label of each highly sensitive signal feature, in which the sum of the Euclidean distance between the training samples and the testing sample is the miner.

Assuming that the training dataset for the highly sensitive signal feature **X***_n_* can be expressed as **N**, the training dataset **N** can be divided into the undamaged cluster and the damaged cluster, thus **N** = {**N**^1^, **N**^2^}. The Euclidean distance $d_{q}^{p}$ between the testing sample *q* and the cluster **N***^p^* can be obtained as follows:

(5) $$d_{q}^{p}=\sqrt{\sum {\left(N^{p}-q\right)}^{2}}$$

Then the state label’s predicted result λ(*q*) of the testing sample *q* can be given by the following:

(6) $$\lambda (q)={\arg\;\min}_{p=1,2}\;d_{q}^{p}$$

where the function arg(.) means that the testing sample’s state label is consistent with the cluster.

### 2.4. Damage Identification Process Based on Highly Sensitive Features and Distance Analysis

The damage identification process based on highly sensitive signal features and distance analysis algorithm includes two procedures: the feature extraction and reduction and the damage identification.

Feature extraction and reduction procedure: Firstly, Lamb wave signals are obtained under different structural health states, including the baseline signals and the respective current signals. Then, 15-dimensional signal features are extracted in the time domain, the frequency domain and the time–frequency domain. Furthermore, the orthogonal signal features are reduced from 15-dimensional signal features based on the Pearson correlation coefficient and cluster analysis algorithm. Finally, the highly sensitive signal features are retained and assigned as the training dataset.

Damage identification procedure: Once a new monitoring experimental situation has occurred, the highly sensitive signal features can be extracted based on the baseline signals and the new current signals of all monitoring paths. Then, the orthogonal and highly sensitive signal features are assigned as the testing dataset. With the training dataset and testing dataset, the distance analysis algorithm is applied to identify the damage. Finally, the damage identification results of different highly sensitive signal features are obtained separately.

## 3. Evidence Theory

### 3.1. Brief Review of Evidence Theory

The damage monitoring information from the single signal feature is incomplete and inaccurate, and the damage identification results of different signal features are likely to be contradictory. Multi-source information fusion theory achieves information fusion based on the uncertain information to obtain the consistent interpretation or description framework of the target object, which has the great significance in improving the accuracy of identification.

According to the information levels, the information fusion methods can be divided into detection-level fusion methods, location-level fusion methods, target recognition-level fusion methods, situation assessment methods and threat assessment methods [29]. The target recognition level fusion methods can be further divided into data-level fusion, feature-level fusion and decision-level fusion.

Evidence theory is proposed by Dempster, and developed by Shafer, thus the evidence theory is also named the D-S evidence theory, and is a mathematical tool to deal with incomplete, uncertain and inaccurate information, widely used in decision-level fusion [30].

Assuming that **U** is the identification framework, function 2**^U^**→[0,1] satisfies the below conditions:

$$\begin{array}{l} (1)\;e(\phi )=0;\\ (2)\;\sum_{\lambda \subset U}e(\lambda )=1; \end{array}$$

where *e*(*λ*) is the BPA of the proposition λ.

*λ_m_* is the state label’s predicted result of the highly sensitive signal features vector **X***_m_* and *λ_l_* is the state label’s predicted result of the highly sensitive signal features vector **X***_l_*. Assuming *e*_1_ and *e*_2_ are the two independent BPAs, the fusion BPA of the proposition *Q* by fusing *e*_1_ and *e*_2_ based on Dempster combination rules can be obtained by the following:

(7) $$e_{1}⊕e_{2}(Q)=\left\{\begin{array}{cc} \frac{\sum_{\lambda_{m}\cap \lambda_{l}=Q}e_{1}(\lambda_{m})e_{2}(\lambda_{l})}{1-D} & Q\neq \phi \\ 0 & Q=\phi \end{array}\right.$$

where *D* is the inconsistency factor, and can be calculated as follows:

(8) $$D=\sum_{\lambda_{l}\cap \lambda_{m}=\phi }e_{1}(\lambda_{m})e_{2}(\lambda_{l})$$

The evidences can be fused in pairs for multiple evidences based on the associative law and commutativity of Dempster combination rules.

### 3.2. The BPA Based on Euclidean Distance

The distance analysis method can obtain the damage identification result of the testing sample for every highly sensitive signal feature without the corresponding BPA. Considering that the probability of the testing sample belonging to this cluster is greater the closer the testing sample is to this cluster, the BPA of the damage identification result for the testing sample is defined as the deviation of the normalized sum for the distances between the testing sample to the damaged cluster and the undamaged cluster to 1, which can be calculated as follows:

(9) $$e(\lambda_{m})=1-\frac{d_{m}^{p}}{\sum_{p}d_{m}^{p}}(p=1,2)$$

where *λ_m_* is the state label’s predicted result, whose value is 0 or 1, representing, respectively, the undamaged label or the damaged label.

### 3.3. The Damage Fusion Identification Process Based on Distance Analysis and Evidence Theory

**Figure 1:**
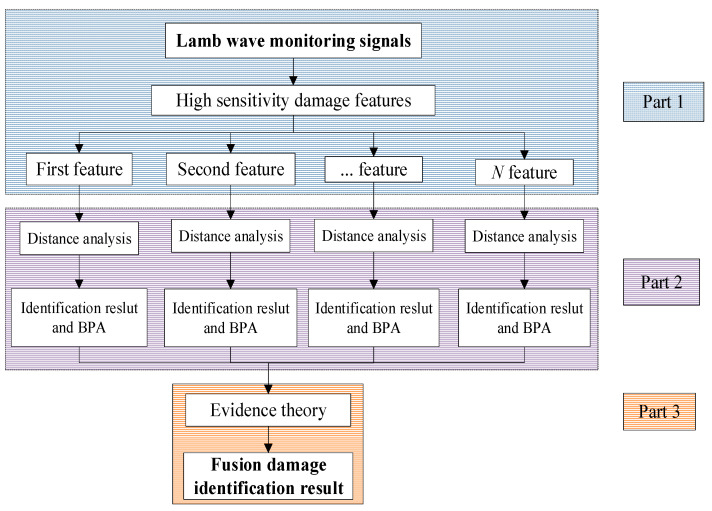
The damage fusion identification process based on distance analysis and evidence theory.

The damage fusion identification method based on distance analysis and evidence theory is proposed, as shown in Figure 1, which includes three parts, namely feature extraction, damage identification and the fusion results. Part 1 extracts 15-dimensional signal features from the time domain, the frequency domain and the time–frequency domain based on LW monitoring signals, and reduces features dimensions based on the Pearson correlation coefficient and cluster analysis in order to retain the orthogonal and highly sensitive signal features. Part 2 obtains the respective damage identification results of the highly sensitive signal features based on the distance analysis algorithm and obtains the corresponding BPAs of the respective damage identification results based on Euclidean distance. Part 3 fuses the damage identification results of the highly sensitive signal features using evidence theory and acquires the consistent damage identification result.

## 4. Experimental Validation on Composite Laminate Panels

### 4.1. Specimen and Experimental Setup

**Figure 2:**
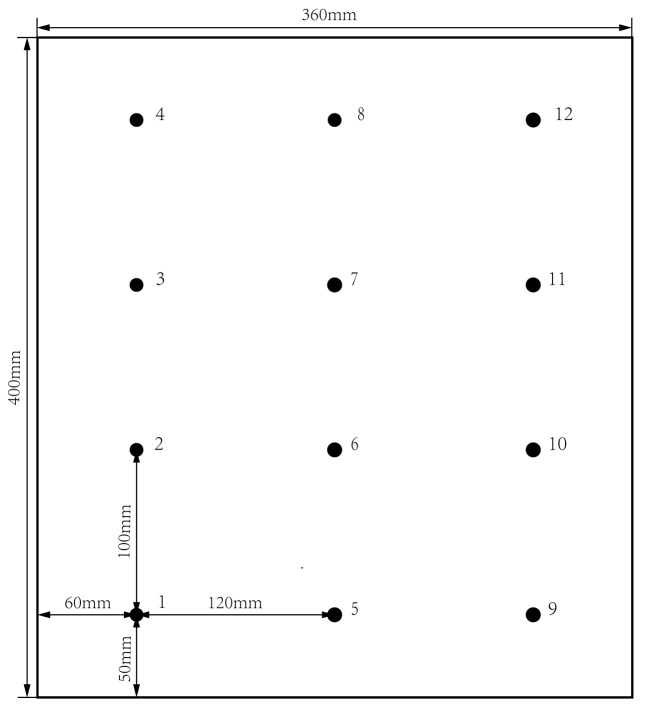
Schematic of composite laminate panel and PZT sensors placement.

**Figure 3:**
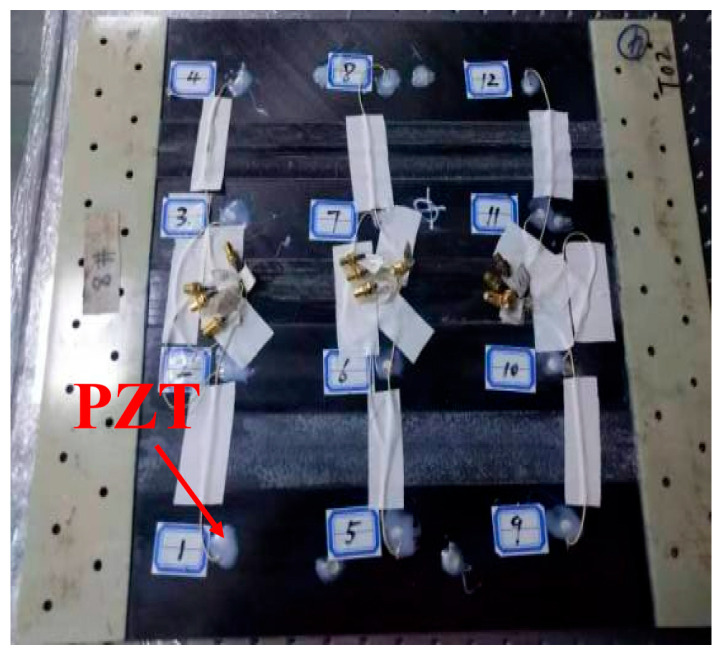
Composites laminate panel and adhesive PZT.

Impact tests on ten specimens are performed to validate the proposed method. These specimens are manufactured using T300/BA9916 composite laminates panels of [0/90/±45] s, labeled from S1 to S10. Twelve lead zirconate titanate (PZT) sensors P51 were bonded on each panel with GLEIHOW302 adhesive with the horizontal distance of 120 mm and the vertical distance of 100 mm. The dimensions of PZT sensors are 8 mm in diameter and 0.45 mm in thickness. The dimensions of the composite laminate panel and PZT sensors placement are shown in Figure 2. One specimen with PZT sensors adhered to it is shown in Figure 3.

**Figure 4:**
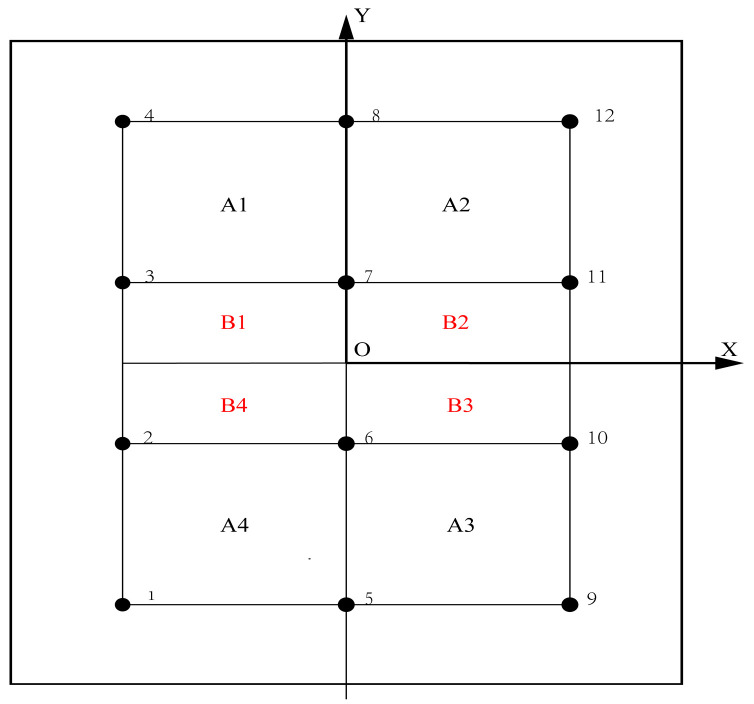
Distribution of secondary monitoring areas.

According to the symmetries of the specimen and the monitoring network, the area encompassed by PZTs was divided into two independent parts, namely area A and area B, in order to make the impact positions more representative. Furthermore, in order to distinguish between the symmetry monitoring areas, the eight secondary symmetry monitoring areas were individually named A1, A2, A3, A4 and B1, B2, B3, B4. The distribution of these secondary monitoring areas with the coordinate system is shown in Figure 4.

The 151 impact positions of ten specimens decided by the random number were allocated into eight secondary monitoring areas. The drop hammer impact device was used to induce the impact damage of diverse areas into specimens by adjusting the height and mass of the drop hammer in order to change the impact energy. The damage area of impact damages was tested with the ultrasonic C-scan equipment and used to represent the damage severity. Three impact tests with various impact positions and damage areas in the A2 secondary monitoring area on specimen S1 are listed in Table 2. Before and after the impact test, the integrated SHM system was used to acquire the baseline signals and current signals of all monitoring paths. The excitation signal was a five-cycle tone burst modulated by a Hanning window with a center frequency of 90 kHz, the sampling rate for the monitored signals was set to 10 MHz and the sampling length was set to 0.5 ms.

The procedure of each damage monitoring experiment is as follows:

(1) Baseline signals and current signals of all monitoring paths were obtained before and after the impact damage was introduced;

(2) The damage area of each impact test was acquired by the ultrasonic C-scan;

(3) The 15-dimensional signal features between the baseline signals and current signals were extracted;

(4) The orthogonal and highly sensitive signal features were retained by reducing feature dimensions based on the Pearson correlation coefficient and cluster analysis;

(5) The damage identification result was obtained based on the distance analysis algorithm.

Considering the severity of the signal’s length to the damage area [31], the proposed damage identification method is only used to identify damages with areas of above 200 mm^2^. Samples whose damage areas are above 200 mm^2^ are labeled as damaged, and samples whose damage areas are below 200 mm^2^ are labeled as undamaged. The max signal feature of six monitoring paths is selected as 1 sample, and 151 samples contain 65 damaged samples.

### 4.2. Highly Sensitive Signal Features

**Figure 5:**
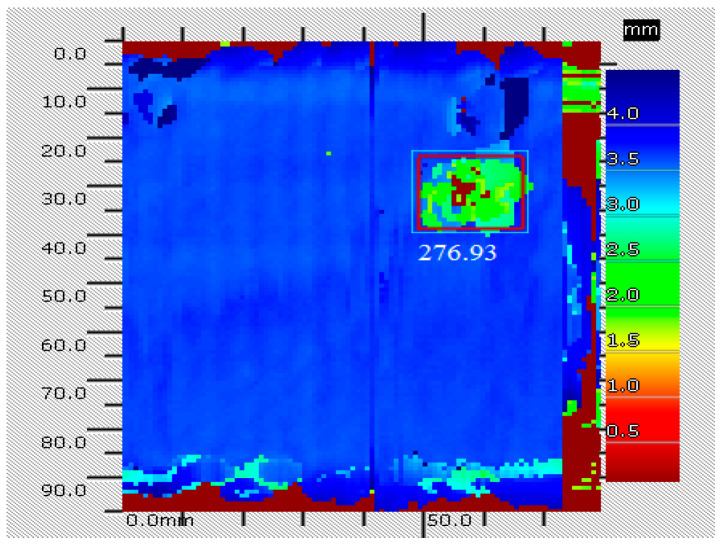
The non-destructive test result of the A2 secondary area by the ultrasonic C-scan.

**Figure 6:**
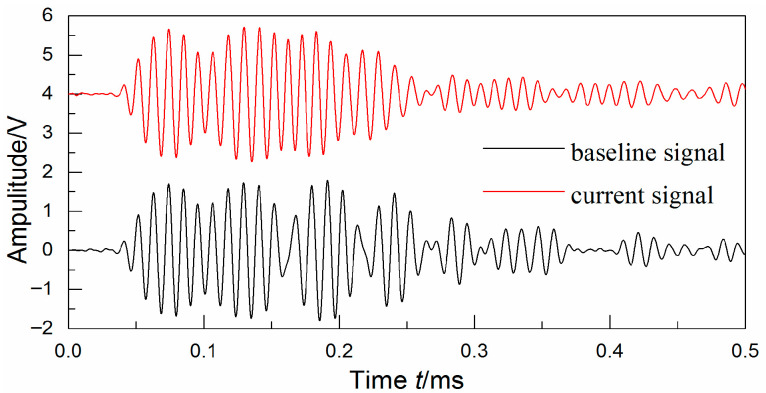
The baseline and current signals of the 7–12 monitoring path with 276.93 mm^2^ delamination.

Figure 5 shows the non-destructive test result of the A2 secondary area by the ultrasonic C-scan in position (118 mm, 117 mm) with 27 J impact energy, which introduced 276.93 mm^2^ delamination damage into the specimen S1. Figure 6 shows the baseline and current signals of the 7–12 monitoring path with 276.93 mm^2^ delamination. The introduced delamination damage has an effect on the monitoring signals, including the appearance of the damage scattering signal and the change in the boundary reflecting signal. To include the damage scattering signals and obtain features more sensitive to the damage, the start time *t*_1_ and the stop time *t*_2_ corresponding to the selected signal’s time window were set to 0.1 ms and 0.16 ms. And the start frequency *w*_1_ and the stop frequency *w*_2_ were set to 70 kHz and 110 kHz.

**Figure 7:**
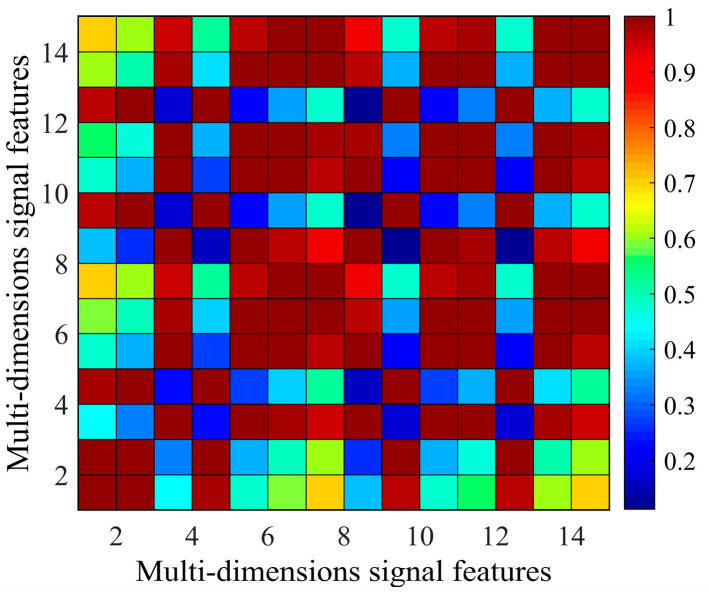
The Pearson correlation coefficient matrix of 15-dimensional signal features.

The 15-dimensional signal features of the 7–12 monitoring path are extracted under three impact tests, and the Pearson correlation coefficients of these signal features are calculated. The maximum of the Pearson correlation coefficient is 1, which indicates a fully positive correlation between two signal features and means the same change of two signal features in response to structural health states. The minimum of the Pearson correlation coefficient is −1, which indicates a fully negative correlation between two signal features and means an adverse change of two signal features in response to structural health states. If the Pearson coefficient is 0, it indicates the non-correlation between two signal features and means that they are independent. Figure 7 shows the absolute Pearson correlation coefficient matrix of 15-dimensional signal features. The maximum absolute Pearson correlation coefficient of 15-dimensional signal features is 1, and the minimum absolute Pearson coefficient is 0.1114. Based on cluster analysis with the threshold *δ* set to 0.9850, 15-dimensional signal features are assembled as four clusters, namely *C*_1_ = {*DI*_1_, *DI*_2_, *DI*wavelet}, *C*_2_ = {*DI*_3_, *SDCC*, *DI*varience, *NCM*, *SSS*}, *C*_3_ = {*DI*_4_, *SDT*, *SDS*} and *C*_4_ = {*DIRMS*, *DIRMSD*, *DI*_5_, *DI*_6_}.

**Figure 8:**
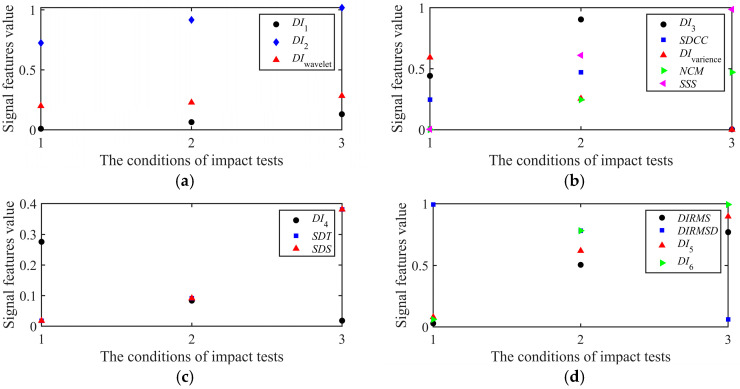
The signal features in four clusters under three impact tests: (**a**) **C**_1_, (**b**) **C**_2_, (**c**) **C**_3_ and (**d**) **C**_4_.

Then, the signal features in four clusters under three impact tests are shown in Figure 8. The sensitivity **Z** of the signal features in four clusters based on Equation (5) is shown in Table 3, representing the sensitivity of signal features in four clusters to the change in structural health states. The signal feature is higher with the larger damage area for all signal features. However, the sensitivity of different signal features to structural health states is different. For example, in the cluster *C*_3_, *SDT* and *SDS* are consistent with each other, but *DI*_4_ has the larger Z, meaning a higher sensitivity to the change in structural health states, and *DI*_4_ can be chosen as one of highly sensitive signal features. Finally, according to the sensitivity of signal features in four clusters under three impact tests, the signal features set {*DI*_2_, *SSS*, *DI*_4_, *DI*_6_} is chosen as the optional and independent highly sensitive signal features set after the dimensional reduction, which can be used to identify the structural damage. The highly sensitive signal features all reflect the signal’s energy in the time domain or the frequency domain, identifying that the signal’s energy-based features, considering both the amplitude and phase changes in the time and frequency domain, are more sensitive to the delamination damage. And in the highly sensitive signal features set, *DI*_6_ is the most sensitive signal feature to the change in structural health states.

### 4.3. Damage Identification Results of Highly Sensitive Signal Features

Firstly, 151 samples were randomly divided into the training dataset and the testing dataset with the ratio of 1:1, and the division of damaged and undamaged samples is shown in Table 4. The training dataset for four highly sensitive signal features, namely *DI*_2_, *SSS*, *DI*_4_ and *DI*_6_, were all divided into the damaged cluster and the undamaged cluster. The state label’s predicted result of each testing sample was obtained based on the Euclidean distance between the testing sample and the damaged or undamaged cluster for four highly sensitive signal features.

**Figure 9:**
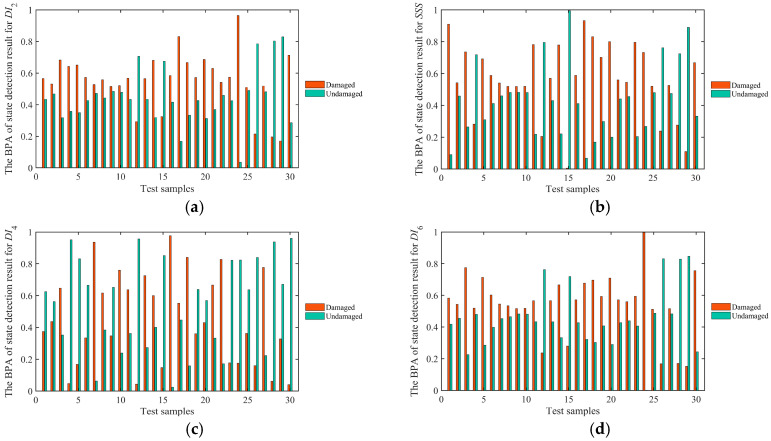
The BPAs of 30 testing samples for four highly sensitive signal features: (**a**) *DI*_2_, (**b**) *SSS*, (**c**) *DI*_4_ and (**d**) *DI*_6_.

Then, the BPA of the detection results of the structural states of the testing sample for four highly sensitive signal features was obtained by computing the Euclidean distance. The BPAs of the states detection results (damaged or undamaged) for 30 testing samples are shown in Figure 9, in which the larger BPA implies the higher probability of the state detection result. As shown in Figure 9, the damage identification capabilities of four highly sensitive signal features are different, thus the damage identification results of some testing samples for four highly sensitive signal features are contradictory.

Finally, the state label’s predicted results of all testing samples for four highly sensitive signal features are shown in Table 5. *A* is the number of the correctly predicted true damaged testing samples. *B* is the number of the incorrectly predicted true damaged testing samples. *C* is the number of the incorrectly predicted true undamaged testing samples. *D* is the number of the correctly predicted true undamaged testing samples. To comprehensively evaluate the classification performance of the proposed method, the classification accuracy, the false alarm rate and the missing alarm rate [32] are all used. The classification accuracy is the proportion of the sum of the correctly predicted true damaged samples and the correctly predicted undamaged samples to the total samples, equivalent to (*A* + *D*)/(*A* + *B* + *C* + *D*). The false alarm rate is the proportion of the wrong predicted true undamaged samples to the total predicted damaged samples, equivalent to *C*/(*C* + *A*). The missing alarm rate is the proportion of the wrong predicted true damaged samples to the total true damaged samples, equivalent to *B*/(*B* + *A*). As shown in Table 5, *DI*_6_ shows the highest classification accuracy and the lowest false alarm rate and missing alarm rate, meaning the optimum damage identification capability, which is consistent with the highest sensitivity of *DI*_6_. But *SSS* has the lowest classification accuracy and the highest false alarm rate and missing alarm rate, meaning the worst damage identification capability, which is consistent with the lowest sensitivity of *SSS*. *DI*_4_ and *DI*_2_ have a higher classification accuracy, lower false alarm rate and missing alarm rate than *SSS*. The damage identification capabilities for four highly sensitive signal features are inconsistent with each other.

### 4.4. Fused Damage Identification Results and Discussion

Based on the BPAs of 76 testing samples for four highly sensitive signal features, the damage fusion identification results were obtained with three decision-level fusions based on evidence theory. The fusion process of two testing samples is shown in Table 6. As shown in Table 6, with more fusion procedures, the uncertainty of the predicted result is lower. And the probability assignments for each highly sensitive signal feature will influence the fusion probability. The fusion predicted results of 76 testing samples were acquired and are shown in Table 7. As shown in Table 7, the damage fusion identification results based on distance analysis and evidence theory are more accurate and reliable than the respective results of four highly sensitive signal features. The classification accuracy of 76 testing samples is higher than 85%, the false alarm rate is lower than 25% and the missing alarm rate is lower than 15%, based on the proposed damage fusion identification method.

## 5. Conclusions

The damage fusion identification method based on distance analysis and evidence theory is proposed in this paper, and the method is used to fuse the respective damage identification results of four highly sensitive signal features in order to obtain the consistent identification results. In the proposed method, four highly sensitive signal features are firstly retained based on the Pearson correlation coefficient and cluster analysis, by reducing 15-dimensional signal features extracted from the time domain, the frequency domain and the time–frequency domain. Then, the respective damage identification results of four highly sensitive signal features are acquired based on distance analysis, and the BPAs of the identification results are obtained by computing the Euclidean distance. Finally, the damage fusion identification result is acquired by fusing the BPAs of the damage identification results for four highly sensitive signal features with the decision-level fusion method, based on evidence theory. The effectiveness of the proposed damage identification method was assessed by identifying impact damages under different locations with different areas on ten composite laminate panels. The results show that the damage fusion identification method can accurately identify the impact damage with high probability, and the classification accuracy of 76 testing samples is above 85%, the false alarm rate is lower than 25% and the missing alarm rate is lower than 15%. The proposed damage fusion identification method presents a superior accuracy and probability for the damage identification of composite laminate panels.

The proposed method is a damage identification method for composite laminate panels, which is only able to accurately detect the delamination damage with areas above 200 mm^2^, but unable to detect small damage cases or further evaluate damage. Therefore, in future research, the small damage detection ability needs to be investigated. Moreover, the small damage location and quantification monitoring methods also need further study.

## Figures and Tables

**Table 1:** 15-dimensional signal features extraction.

Column	Signal Features	Description
1	Peak-to-peak amplitude	$DI_{1}=\left|A_{b}-A_{d}\right|$
2	Energy ratio in the time domain	$DI_{2}=\int_{t_{1}}^{t_{2}}b^{2}(t)dt/\int_{t_{1}}^{t_{2}}d^{2}(t)dt$
3	Scatter signal difference in the time domain	$DI_{3}=\left(\int_{t_{1}}^{t_{2}}{\left(d(t)-b(t)\right)}^{2}dt\right)/\left(\int_{t_{1}}^{t_{2}}{\left(d(t)\right)}^{2}dt\right)$
4	Energy change ratio in the time domain	$DI_{4}=\left|\int_{t_{1}}^{t_{2}}{\left(d(t)\right)}^{2}dt-\int_{t_{1}}^{t_{2}}{\left(b(t)\right)}^{2}dt\right|/\left|\int_{t_{1}}^{t_{2}}{\left(d(t)\right)}^{2}dt\right|$
5	Signal difference correlation coefficient	$SDCC=1-\rho_{bd}$
6	Root mean square	$DIRMS=\sqrt{\frac{1}{t_{2}-t_{1}}\int_{t_{1}}^{t_{2}}{\left(d(t)-b(t)\right)}^{2}dt}$
7	Root mean square deviation	$DIRMSD=\sqrt{\int_{t_{1}}^{t_{2}}{\left(d(t)-b(t)\right)}^{2}dt/\int_{t_{1}}^{t_{2}}{\left(b(t)\right)}^{2}dt}$
8	Variance damage index	$DI\mathrm{variance}=1/\left(t_{2}-t_{1}\right)\sqrt{\int_{t_{1}}^{t_{2}}{\left(\left(d(t)-b(t)\right)-\left(\bar{d(t)-b(t)}\right)\right)}^{2}dt}$
9	Signal difference in the time domain	$SDT=\left|\int_{t_{1}}^{t_{2}}{\left(b(t)\right)}^{2}dt-\int_{t_{1}}^{t_{2}}{\left(d(t)\right)}^{2}dt\right|/\left(\int_{t_{1}}^{t_{2}}{\left(b(t)\right)}^{2}dt\right)$
10	Normalized correlation moment	$NCM=\left|\int_{\tau =t_{1}}^{\tau =t_{2}}\tau^{2}r_{bb}(\tau )d\tau -\int_{\tau =t_{1}}^{\tau =t_{2}}\tau^{2}r_{bd}(\tau )d\tau \right|/\left|\int_{\tau =t_{1}}^{\tau =t_{2}}\tau^{2}r_{bb}(\tau )d\tau \right|$
11	Energy ratio in the frequency domain	$SSS=\left(\int_{w_{1}}^{w_{2}}{\left(b\left(w\right)-d\left(w\right)\right)}^{2}dw\right)/\left(\int_{w_{1}}^{w_{2}}{\left(b\left(w\right)\right)}^{2}dw\right)$
12	Signal difference in the frequency domain	$SDS=\left|\int_{w_{1}}^{w_{2}}{\left(b(w)\right)}^{2}dw-\int_{w_{1}}^{w_{2}}{\left(d(w)\right)}^{2}dw\right|/\left|\int_{w_{1}}^{w_{2}}{\left(b\left(w\right)\right)}^{2}dw\right|$
13	Scatter signal difference in the frequency domain	$DI_{5}=\left(\int_{w_{1}}^{w_{2}}{\left(d(w)-b(w)\right)}^{2}dw\right)/\left(\int_{w_{1}}^{w_{2}}{\left(d\left(w\right)\right)}^{2}dw\right)$
14	Energy change ratio in the frequency domain	$DI_{6}=\sqrt{\left|\int_{w_{1}}^{w_{2}}{\left(d(w)\right)}^{2}dw-\int_{w_{1}}^{w_{2}}{\left(b(w)\right)}^{2}dw\right|/\left|\int_{w_{1}}^{w_{2}}{\left(d\left(w\right)\right)}^{2}dw\right|}$
15	Wavelet packet decomposition coefficient	$DI\mathrm{wavelet}(i,j)=\langle \left(b(t)-d(t)),\psi (t\right)\rangle$

Where *A_b_* and *A_d_* are the max amplitude of the baseline signals *b*(*t*) and the current signals *d*(*t*) obtained by Hilbert transform. *t*_1_ and *t*_2_ are the start and stop times that correspond to the selected signal’s time window. *ρ_bd_* is the correlation coefficient between *b*(*t*) and *d*(*t*). $\bar{d(t)-b(t)}$ is the average of the scatter signals in the selected signal’s time window. *r_bb_*(*τ*) is the self-correlation coefficient of *b*(*t*) and *r_bd_*(*τ*) is the correlation coefficient between *b*(*t*) and *d*(*t*). $b(w)=\int_{w_{1}}^{w_{2}}b(t)e^{-j\omega t}dw$ and $d(w)=\int_{w_{1}}^{w_{2}}d(t)e^{-j\omega t}dw$. *w*_1_ and *w*_2_ are the start and stop frequency that correspond to the selected frequency spectrum window. *ψ*(*t*) is the wavelet mother function which is db6 wavelet mother function in this paper. *DI*wavelet(*i*, *j*) is the wavelet packet decomposition coefficient of the scatter signals decomposed with *ψ*(*t*), in which *i* is decomposition scale and *j* is the frequency band. In this paper, *i* = 3 and *j* = 6.

**Table 2:** Impact positions and damage areas of three impact tests in A2 secondary monitoring area.

Condition	Impact Position (x,y)/mm	Damage Area/mm^2^
1	(40, 116)	163.20
2	(54, 118)	239.36
3	(118, 117)	276.93

**Table 3:** The sensitivity **Z** of signal features in four clusters under three impact tests.

**Cluster C_1_**	**Sensitivity Z**	**Cluster C_2_**	**Sensitivity Z**
*DI*_1_	2.6561	*DI*_3_	0.0269
*DI*_2_	4.7723	*SDCC*	0.0426
*DI*wavelet	2.0597	*DI*varience	3.6538
		*NCM*	3.1780
		*SSS*	4.2212
**Cluster C_3_**	**Sensitivity Z**	**Cluster C_4_**	**Sensitivity Z**
*DI*_4_	6.8687	*DIRMS*	4.5965
*SDT*	1.5074	*DIRMSD*	1.5894
*SDS*	1.5074	*DI*_5_	4.8832
		*DI*_6_	7.7861

**Table 4:** The division of damaged and undamaged samples for the training and testing dataset.

Dataset	Damaged Samples	Undamaged Samples
Training dataset	34	41
Testing dataset	31	45

**Table 5:** The state label’s predicted results of 76 testing samples for four highly sensitive signal features.

Highly Sensitive Signal Features		Predicted	Damaged	Undamaged	Classification Accuracy	False Alarm Rate	Missing Alarm Rate
	True						
*DI*_2_	damaged		*A* = 21	*B* = 10	59.21%	50.00%	32.26%
	undamaged		*C* = 21	*D* = 24			
*SSS*	damaged		*A* = 17	*B* = 14	53.95%	55.26%	45.16%
	undamaged		*C* = 21	*D* = 24			
*DI*_4_	damaged		*A* = 22	*B* = 9	61.84%	47.62%	29.03%
	undamaged		*C* = 20	*D* = 25			
*DI*_6_	damaged		*A* = 22	*B* = 9	73.68%	33.33%	29.03%
	undamaged		*C* = 11	*D* = 34			

**Table 6:** The fusion process of two testing samples based on evidence theory.

Testing Sample 1	The BPA of Damaged Label	The BPA of Undamaged Label	Testing Sample 2	The BPA of Damaged Label	The BPA of Undamaged Label
*DI*_2_ ⊕ *SSS*	0.8974	0.1026	*DI*_2_ ⊕ *SSS*	0.1572	0.8428
*DI*_2_ ⊕ *SSS* ⊕ *DI*_4_	0.8687	0.1313	*DI*_2_ ⊕ *SSS* ⊕ *DI*_4_	0.1024	0.8976
*DI*_2_ ⊕ *SSS* ⊕ *DI*_4_ ⊕ *DI*_6_	0.9417	0.05830	*DI*_2_ ⊕ *SSS* ⊕ *DI*_4_ ⊕ *DI*_6_	0.0622	0.9378
Predicted result	damaged		Predicted result	undamaged	

**Table 7:** The fusion predicted results of 76 testing samples.

	Predicted	Damaged	Undamaged	Classification Accuracy	False Alarm Rate	Missing Alarm Rate
True						
damaged		*A* = 27	*B* = 4	85.53%	20.59%	12.90%
undamaged		*C* = 7	*D* = 38			

## Data Availability

The datasets presented in this article are not readily available due to technical limitations.
